# Genome-wide association mapping reveals stable genomic regions associated with fruit quality traits in tomato (*Solanum lycopersicum* L.)

**DOI:** 10.3389/fpls.2026.1972937

**Published:** 2026-10-15

**Authors:** Raja Sekhar Srungarapu, Devi R. Kandel, Mohan Singh Rajkumar, Kranthi K. Mandadi, Kevin Crosby, Carlos A. Avila

**Affiliations:** 1 Texas A&M AgriLife Research and Extension Center, Weslaco, TX, United States; 2 Langston University, Department of Agriculture and Natural Resources, Langston, OK, United States; 3 Department of Plant Pathology and Microbiology, Texas A&M University, College Station, TX, United States; 4 Department of Horticultural Sciences, Texas A&M University, College Station, TX, United States

**Keywords:** fruit color, fruit quality, fruit weight, genome-wide association study, skim sequencing, SNPS, soluble solids, tomato

## Abstract

Fruit quality has become a primary breeding objective in tomato because it determines consumer acceptance, processing efficiency, and market value. However, quality-related traits are quantitatively inherited and strongly influenced by environmental conditions, making genetic improvement challenging. In this study, genome-wide association studies (GWAS) was conducted to elucidate the genetic architecture of six economically important fruit quality traits, including luminosity, hue, chroma, soluble solids content (°Brix), titratable acidity, and fruit fresh weight (FW), using a diverse panel of 305 tomato accessions evaluated over two growing seasons. Substantial phenotypic variation was observed for all traits, with significant effects of genotype, year, and genotype × year interaction, indicating that fruit quality is governed by both genetic and environmental factors. Fruit weight, luminosity, hue, chroma, and °Brix exhibited high broad-sense heritability, whereas titratable acidity showed moderate heritability. Correlation analysis revealed strong positive relationships among color-related traits, while °Brix was positively associated with acidity but negatively correlated with fruit color attributes and FW. Skim sequencing generated 4,618 high-quality single nucleotide polymorphisms (SNPs), and GWAS was performed using multiple models. GWAS identified 15 non-redundant marker-trait associations (MTAs) across chromosomes, with several loci consistently detected across statistical models. A prominent locus, SL4.0ch03_12722521, on chromosome 3 was associated with luminosity, hue, and chroma, explaining up to 53.86% of phenotypic variation (PVE), suggesting pleiotropic regulation of fruit color. Stable loci controlling FW and soluble solids were also identified on chromosome 5, explaining up to 52.88% PVE. Candidate genes of significant MTAs included sucrose synthase, sec14p-like phosphatidylinositol transfer protein, bHLH transcription factor, and chitinase genes, indicating potential roles in carbohydrate metabolism, fruit development, cell wall remodeling, pigmentation, and ripening. These findings provide valuable genomic resources for improving fruit quality and accelerating the development of elite tomato cultivars.

## 1 Introduction

Tomato (*Solanum lycopersicum* L.) is one of the world’s most economically important vegetable crops and is cultivated across diverse agroecological regions of the world. In addition to its economic value, tomato represents an important dietary source of vitamins, carotenoids, minerals, phenolic compounds, and antioxidants that contribute significantly to human nutrition and health (Borguini and Ferraz da Silva Torres, 2009; Collins et al., 2022). Beyond its significance as a fresh-market vegetable, tomato is extensively utilized in the processing industry for products such as sauces, juices, pastes, and canned foods (Thakur et al., 1996). Owing to its versatility in fresh consumption and industrial processing, tomato contributes substantially to global food security and the agricultural economy.

Breeding efforts have shifted considerably in recent years from focusing on the development of high-yielding cultivars toward varieties that also offer superior fruit quality, including enhanced flavor, nutritional value, texture, color, and shelf life (Jones and Scott, 1983; Baldwin et al., 2008; Hickey et al., 2019). Fruit quality is a complex trait governed by the interaction of multiple genetic and environmental factors (Beckles, 2012; Lupi et al., 2019). Attributes such as fruit weight, shape, firmness, soluble solids content, acidity, sugar composition, lycopene concentration, carotenoid accumulation, vitamin C content, and fruit color are quantitative in nature and are controlled by numerous genes with varying effects (Frary et al., 2000; Mu et al., 2017; Li et al., 2020). The expression of these traits is further influenced by growing conditions, agronomic practices, and genotype × environment interactions, making genetic improvement particularly challenging through conventional breeding alone (Beckles, 2012; Klee and Tieman, 2013). Moreover, valuable alleles present in traditional cultivars, landraces, and wild relatives remain underutilized in breeding programs despite representing an important reservoir of genetic diversity for quality improvement (Blanca et al., 2015; Liu et al., 2021). Therefore, a deeper understanding of the genetic architecture underlying fruit quality traits is essential for developing tomato cultivars that satisfy both market demands and nutritional requirements.

In the present study, key quality attributes include luminosity, hue, chroma, total soluble solids (°Brix), titratable acidity, and fruit weight (FW). Luminosity, hue, and chroma collectively determine fruit color and visual appeal, which strongly influence consumer acceptability and marketability (Barrett et al., 2010; Thole et al., 2020). Soluble solids, expressed as °Brix, largely reflect sugar accumulation and contribute to sweetness and processing efficiency, whereas titratable acidity determines flavor balance and overall taste perception (Fulton et al., 2002; Causse et al., 2010; Wang et al., 2023). Fruit fresh weight directly affects market grade, yield, and processing efficiency (Tanksley, 2004). Because these traits collectively define commercial quality, they have become primary targets in contemporary tomato improvement programs. Consequently, improving fruit quality has become one of the foremost objectives of modern tomato breeding programs.

The rapid advancement of next-generation sequencing technologies has transformed the genetic analysis of complex quantitative traits in crop species (Agarwal et al., 2008; Nordborg and Weigel, 2008). In tomato, skim sequencing provides an efficient approach for generating genome-wide single nucleotide polymorphism (SNP) markers suitable for high-resolution genetic analyses (Myles et al., 2009; Kumar et al., 2021). Among the available approaches, genome-wide association studies (GWAS) have emerged as a powerful strategy for dissecting the genetic basis of complex quantitative traits by exploiting historical recombination within diverse germplasm at much higher resolution than traditional biparental linkage mapping (Davey et al., 2011; Huang and Han, 2014). The integration of dense genome-wide SNP markers with advanced statistical models, including FarmCPU, BLINK, MLMM, and SUPER, has greatly improved the ability to detect loci underlying quantitative traits while reducing false-positive associations caused by population structure and kinship (Zhang et al., 2010; Runcie and Crawford, 2019). Nevertheless, differences in model assumptions often lead to variation in the loci detected; therefore, evaluating multiple GWAS models provides greater confidence in identifying stable genomic regions associated with target traits. Identification of robust and reproducible marker-trait associations not only enhances our understanding of the molecular mechanisms controlling fruit quality but also provides practical genomic tools for breeding elite cultivars with improved nutritional value, consumer preference, and postharvest performance. Such genomic information is increasingly important for addressing the dual challenge of meeting growing global food demand while maintaining crop quality under changing climatic conditions (Anderson et al., 2020).

Several GWAS studies have identified genomic regions controlling fruit size, shape, soluble solids, acidity, pigment accumulation, disease resistance, flowering time, and yield-related traits in tomato (Barrett et al., 2010; Zhang et al., 2015; Phan et al., 2019; Thole et al., 2020; Kim et al., 2021; Zhao et al., 2022; Liu et al., 2023b; Kayikci et al., 2025). Nevertheless, the genetic basis of several commercially important fruit quality traits, particularly those describing fruit color (luminosity, hue, and chroma), sweetness (°Brix), acidity, and fruit weight, remains incompletely understood because of their polygenic inheritance, the limited diversity of mapping populations used in previous studies, and the strong influence of environmental variation (Phan et al., 2019; Kayikci et al., 2025). The integration of GWAS with modern genomic resources offers valuable opportunities for accelerating marker-assisted selection, genomic selection, and gene discovery in tomato improvement (Duangjit et al., 2016; Crossa et al., 2017; Hernández-Bautista et al., 2020).

In the present study, 305 genetically diverse tomato accessions were evaluated over two growing seasons for six economically important fruit quality traits, including luminosity, hue, chroma, soluble solids content (°Brix), titratable acidity, and fruit fresh weight. Best linear unbiased estimators (BLUEs) were generated using mixed-model analyses to obtain robust genotype estimates across seasons. GWAS were subsequently conducted using 4,618 high-quality SNP markers generated through skim sequencing and analyzed using multiple single-locus and multi-locus GWAS models. The objectives of this study were to (i) characterize phenotypic variation and genetic relationships among fruit quality traits, (ii) identify stable MTAs across statistical models, and (iii) discover putative candidate genes underlying significant MTAs. The identified genomic regions and candidate genes provide valuable resources for marker-assisted selection and contribute to a better understanding of the genetic mechanisms governing tomato fruit quality.

## 2 Materials and methods

### 2.1 Plant materials and field evaluation

A diverse association panel comprising 305 tomato (*Solanum lycopersicum* L.) accessions representing broad genetic diversity was used to investigate the genetic architecture of fruit quality traits (**Supplementary Table 1**). The panel was evaluated under field conditions over two consecutive spring seasons in 2018 and 2019 at the Texas A&M AgriLife Research and Extension Center in Weslaco, TX, at a latitude of 26° 9 30” N and a longitude of 97° 57’ 43”. Field row plot spacing was 1.2 m between rows and 1.2 m between plots within a row. Each plot consisted of four plants spaced 0.6 m apart. Standard agronomic practices recommended for tomato production, including irrigation, fertilization, pest management, and weed control, were uniformly implemented throughout the experimental period to minimize non-genetic variation. Fruits were harvested at the red-ripe stage, corresponding to commercial maturity, to ensure consistency in phenotypic measurements across accessions and years.

### 2.2 Phenotypic evaluation

Six economically important fruit quality traits were evaluated: luminosity, hue, chroma, total soluble solids (°Brix), titratable acidity (TA), and fruit fresh weight (FW) (**Supplementary Table 2**). These traits were selected because they collectively represent major components of tomato fruit appearance, flavor, and market quality. Fruit color parameters (luminosity, hue, and chroma) were measured on the fruit surface using a calibrated CR-300 chromameter (Konica Minolta Sensing Americas Inc., Ramsey, NJ). Luminosity (L) represented fruit brightness, hue described the dominant fruit color, and chroma quantified color saturation or intensity. Total soluble solids were determined from freshly extracted fruit juice using a digital refractometer, PAL-BX|ACID3 Brix-Acidity Meter (ATAGO Inc., Bellevue, WA) and expressed as °Brix. Titratable acidity was quantified by titration with a 0.1N NaOH solution using the EasyPlus titration device (Mettler Toledo, Columbus, OH) and expressed as a percentage of citric acid equivalents. Fruit weight was calculated by averaging the total weight of sampled fruits divided by the total number of fruits. Weight was measured on a precision electronic balance and expressed in grams (g).

### 2.3 Phenotypic data analysis

Phenotypic analyses were performed separately for each trait using linear mixed models in R version 4.5.3. Prior to statistical analyses, all traits were examined for normality and homogeneity of variance. A Shapiro-Wilk test was used to evaluate normality, with *P* < 0.05 indicating significant deviation from a normal distribution. Skewness was evaluated as an additional measure of distributional asymmetry. Data transformations were based on the combined assessment of Shapiro-Wilk statistics, skewness, and graphical inspection rather than on the *P*-value alone (**Supplementary Table 3**). Since the traits deviated from normality, data transformations were applied before subsequent analyses to satisfy model assumptions (**Supplementary Table 3**). The transformed datasets were used for all statistical analyses and genome-wide association mapping.

Analysis of variance (ANOVA) was conducted using linear mixed models to evaluate the effects of genotype (G), year (Y), and genotype × year (G × Y) interaction, which were treated as random effects, while replication effects were incorporated according to the experimental design. Since the dataset contained unbalanced observations resulting from differences in fruit availability among accessions, best linear unbiased estimators (BLUEs) were estimated across years. BLUE values were subsequently used for genome-wide association analyses because they provide adjusted phenotypic estimates by accounting for environmental variation while preserving genetic effects (Holland and Piepho, 2024).

Descriptive statistics, including minimum, maximum, mean, standard deviation, and frequency distributions, were calculated for each trait. Broad-sense heritability (H²) was estimated for each trait using variance components obtained from the mixed model to quantify the proportion of total phenotypic variation attributable to genetic effects (Robinson et al., 1949). Phenotypic relationships among fruit quality traits were investigated using Pearson’s correlation coefficients, using the “*metan*” package in R. Correlation matrices were visualized as heatmaps to facilitate interpretation of relationships among traits. Frequency distribution histograms overlaid with normal density curves were constructed using the R package “*ggplot2”*. All visualizations, statistical modeling, and box plot styling were executed using the “*ggplot2”* and “*stats”* packages in R 4.5.3.

### 2.4 DNA extraction and skim sequencing

Genomic DNA was extracted from young, healthy leaf tissue collected from each accession of four-week-old seedlings using a modified cetyltrimethylammonium bromide (CTAB) protocol (Allen et al., 2006). DNA quality and quantity were assessed using spectrophotometry and agarose gel electrophoresis prior to sequencing (Life Technologies, Carlsbad, CA).

Genome-wide single nucleotide polymorphism (SNP) markers were generated through skim sequencing, a low-coverage whole-genome sequencing (SkimGBS) approach that enables efficient and cost-effective discovery of genome-wide genetic variations (Golicz et al., 2014). The paired-end 150 bp long reads were processed to remove low-quality reads, adaptor sequences, and primer contamination using fastp (Chen et al., 2018). The filtered high-quality reads (≥20) were mapped to the *Solanum lycopersicum* reference genome (v4.0) using Bowtie2 (v2.2.5) (Langmead and Salzberg, 2012). The uniquely mapped reads were retained by removing the redundant reads using samtools (v1.6) (Li et al., 2009). Variant calling file (VCF) was generated using Freebayes (v1.3.10) (Garrison and Marth, 2012) with the criteria of ≥20 mapping quality and ≥5 reads coverage at each polymorphic site. The output VCF was further processed to filter single-nucleotide polymorphisms (SNPs) using VCFtools (Danecek et al., 2011; Kandel et al., 2019).

To ensure high-quality genotype data, SNP markers were filtered based on quality criteria, including missingness (≥ 30%), minor allele frequency (MAF ≤ 0.05), and heterozygosity (≤ 10%). to ensure reliable marker-trait association analyses. Missing genotypes were imputed using standard imputation procedures implemented within the genotyping pipeline (Browning and Browning, 2007).

Principal component analysis (PCA) was performed using the quality-controlled SNP dataset to characterize major groups of genetic variation among the accessions. The first two principal components (PCs) were used to visualize population structure. Genetic relationships among accessions were assessed using a neighbor-joining (NJ) tree constructed from pairwise genetic distances calculated from the filtered SNP dataset. The resulting tree was visualized as a fan-shaped phylogeny using GAPIT v4.1. Pairwise genetic relatedness among accessions was estimated using the VanRaden method in GAPIT v4.1, and the resulting kinship matrix was used to characterize the relatedness structure of the association panel. Genome-wide LD decay was estimated by calculating pairwise linkage disequilibrium between SNP markers as a function of their physical distance. The relationship between LD and genomic distance was visualized to characterize the extent of marker correlation across the genome.

### 2.5 Genome-wide association analysis

Genome-wide association analyses were performed using the Genome Association and Prediction Integrated Tool, GAPIT v4.1 (Lipka et al., 2012; Wang and Zhang, 2026) implemented in R v4.5.3. Marker trait associations were evaluated by utilizing both the standard multi-step multi-locus framework (Kansas) and the advanced iterative refinement layer (NYC). Within the software architecture, these configurations are designated as the Kansas and NYC output pipelines, respectively. It is worth noting that these names represent specific statistical modeling iterations and visualization styles unique to the software, rather than physical field environments. The BLUE values obtained from multi-year phenotypic analysis were used as adjusted phenotypic inputs. To maximize the robustness of marker detection and compare statistical performance, seven GWAS models were employed, including the General Linear Model (GLM), Mixed Linear Model (MLM), Compressed Mixed Linear Model (CMLM), Settlement of MLM Under Progressively Exclusive Relationship (SUPER), Fixed and Random Model Circulating Probability Unification (FarmCPU), Bayesian-information and Linkage-disequilibrium Iteratively Nested Keyway (BLINK), and Multiple Loci Mixed Model (MLMM) (Zhang et al., 2010; Liu et al., 2016; Huang et al., 2019). Population structure (principal components) and kinship matrices were incorporated into each model to minimize false-positive associations arising from relatedness and population stratification. Manhattan plots and quantile-quantile (Q-Q) plots were generated to evaluate model performance and the distribution of observed versus expected association statistics. Genome-wide significance was assessed using a Bonferroni-adjusted threshold calculated as α/m, where α = 0.05 and m represents the number of SNP markers tested. Accordingly, associations with P-values below the adjusted threshold were considered statistically significant. In addition to statistical significance, associations detected consistently across multiple GWAS models were prioritized as highly reliable candidate loci for fruit quality traits.

### 2.6 Candidate gene identification

To identify putative candidate genes, significant SNPs were anchored to the tomato reference genome (SL4.0). Genes located within a ±50 kb flanking genomic regions of a significantly associated SNP marker were extracted using gene coordinates available in the ITAG 4.0 gff file. Further, annotations/functions of overlapping or proximal genes were examined to identify genes potentially involved in fruit development, carbohydrate metabolism, pigment biosynthesis, cell wall modification, and other biological processes associated with tomato fruit quality.

The phenotypic effects of favorable and unfavorable alleles at significant loci were further evaluated by comparing adjusted mean trait values between alternative allelic classes through a continuous linear regression and one-way Analysis of Variance (ANOVA). These analyses provided insight into the magnitude and direction of allelic effects and their potential utility for marker-assisted breeding.

## 3 Results

### 3.1 Phenotypic variation of fruit quality traits

Substantial phenotypic variation was observed among 305 tomato accessions for all six fruit quality traits evaluated over two growing seasons (**Table 1**). The frequency distribution histograms revealed continuous variation across all measured fruit quality traits, which is characteristic of complex, polygenic inheritance (**Figure 1**). Box plots demonstrated distinct phenotypic shifts for fruit quality traits (**Figure 1**). The wide range of variation was observed for fruit quality traits, with luminosity ranging from 37.45-61.17 in 2018 and 32.85-63.51 in 2019, reflecting differences in surface brightness. Hue values are crucial indicators of overall maturity and green/yellow to red/yellow pigment shifts, ranging from 30.40-91.70 in 2018 and 30.28-95.56 in 2019. Fruit color intensity, represented by chroma, exhibited notable variations ranging from 17.90-54.64 in 2018 and 19.37-57.94 in 2019. Brix (1.00-7.80%) in 2018 and 2.20-7.60 in 2019, TA (0.10-3.05%) in 2018 and 0.15-2.99 in 2019, and FW (13.60-409.90 g) in 2018 and 9.20-336.60 g in 2019. This variation indicates the presence of considerable genetic diversity within the association panel, providing a suitable foundation for genome-wide association analysis.

**Table 1:** Descriptive statistics of tomato fruit quality traits across two growing seasons.

Trait	Year	Mean	Standard_Dev	Minimum	Maximum
Acidity	2018	0.88	0.39	0.10	3.05
Acidity	2019	0.65	0.36	0.15	2.99
Brix	2018	4.98	0.84	1.00	7.80
Brix	2019	4.66	0.74	2.20	7.60
Chroma	2018	36.74	6.18	17.90	54.64
Chroma	2019	39.43	6.71	19.37	57.94
FW	2018	146.87	71.35	13.60	409.90
FW	2019	125.76	61.83	9.20	336.60
Hue	2018	49.02	10.67	30.40	91.70
Hue	2019	52.53	10.28	30.28	95.56
Luminosity	2018	44.46	3.91	37.45	61.17
Luminosity	2019	47.28	4.36	32.85	63.51

**Figure 1:**
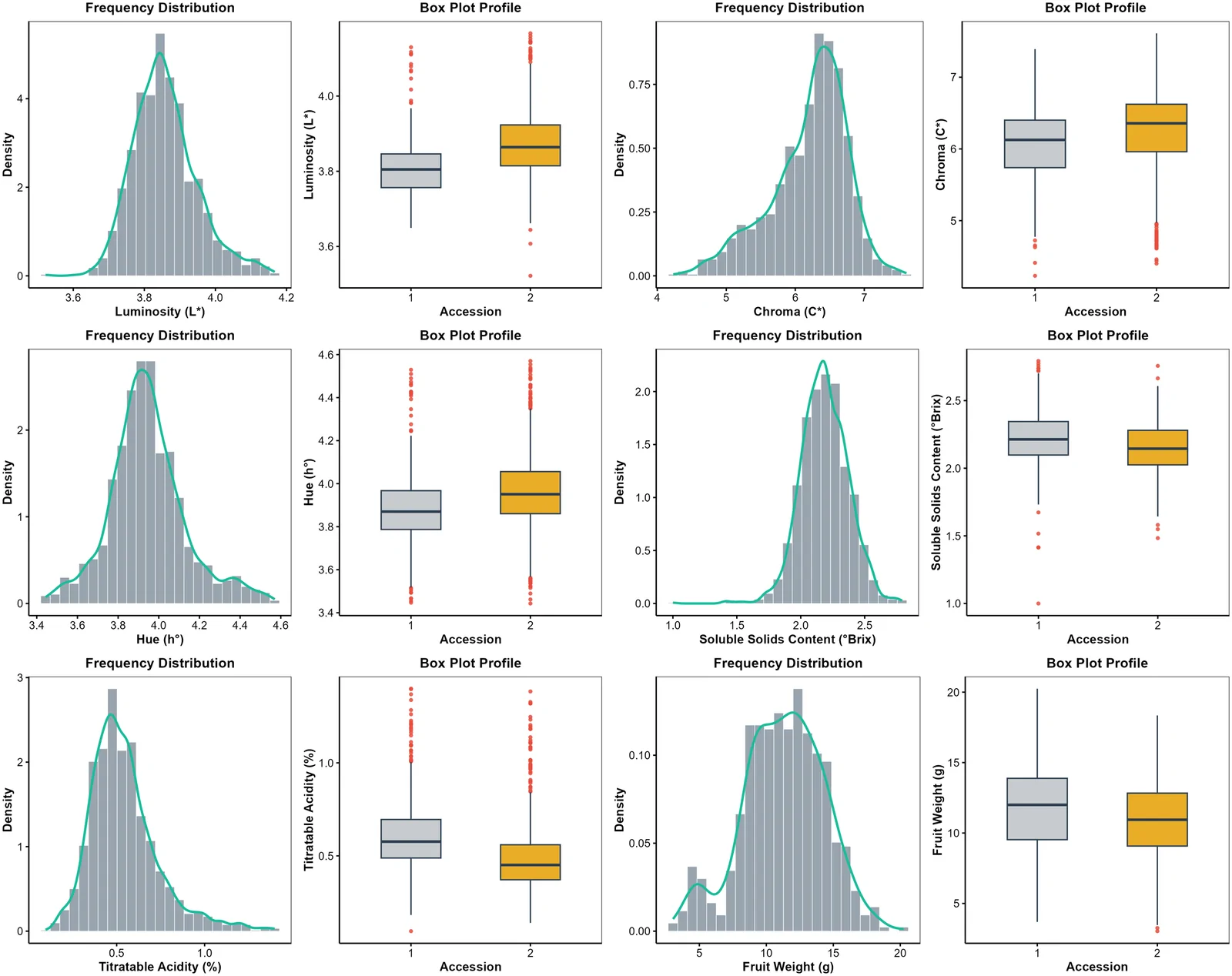
Comprehensive phenotypic characterization of tomato fruit quality traits. Left: Continuous frequency distribution histograms. Right: Year-stratified box plots highlighting population-wide median variance and outlier thresholds.

Analysis of variance (ANOVA) revealed highly significant (P < 0.001) effects of genotype (G), year (Y), and genotype × year (G × Y) interaction for all fruit quality traits under the study (**Table 2**). These results demonstrate the existence of substantial genetic variability among accessions while also highlighting the influence of environmental conditions on fruit quality expression.

**Table 2:** ANOVA for tomato fruit quality traits across two seasons.

Trait	Source of variation	Df	Sum of squares	Mean square	F-statistic	P-value	Sig.
Luminosity	Genotype	304	6.929	0.023	12.304	< 0.001	***
	Year	1	0.790	0.790	426.230	< 0.001	***
	Genotype: Year	296	0.965	0.003	1.759	< 0.001	***
	Residuals	601	1.113	0.002			ns
Chroma	Genotype	304	306.366	1.008	23.840	< 0.001	***
	Year	1	10.086	10.086	238.602	< 0.001	***
	Genotype: Year	296	29.120	0.098	2.327	< 0.001	***
	Residuals	601	25.406	0.042			ns
Hue	Genotype	304	34.822	0.115	20.370	< 0.001	***
	Year	1	1.039	1.039	184.841	< 0.001	***
	Genotype: Year	296	3.048	0.010	1.831	< 0.001	***
	Residuals	601	3.380	0.006			ns
Brix	Genotype	304	25.352	0.083	4.791	< 0.001	***
	Year	1	1.731	1.731	99.476	< 0.001	***
	Genotype: Year	290	11.485	0.040	2.275	< 0.001	***
	Residuals	1,093	19.024	0.017			ns
Acidity	Genotype	304	15.580	0.051	1.910	< 0.001	***
	Year	1	6.280	6.280	234.084	< 0.001	***
	Genotype: Year	290	11.993	0.041	1.541	< 0.001	***
	Residuals	1,092	29.298	0.027			ns

***Significance at 0.001 level; ns, non-significance.

Broad-sense heritability (H²) estimates varied among fruit quality traits, indicating differences in the relative contribution of genetic and environmental factors (**Supplementary Table 4**). Fruit weight exhibited the highest heritability (0.97), suggesting strong genetic control and selection efficiency. Luminosity, Hue, chroma, and Brix displayed high heritability (H² > 0.60). Acidity showed moderate heritability (H² < 0.30), indicating a greater influence of environmental conditions (**Supplementary Table 4**). The high broad-sense heritability estimates observed for several traits indicate that a substantial proportion of the observed phenotypic variation was attributable to genetic differences among accessions under the conditions evaluated. Because broad-sense heritability includes both additive and non-additive genetic effects, these estimates should not be interpreted as direct evidence of additive or near-additive inheritance.

Correlation analysis revealed significant relationships among fruit quality traits in this study (**Figure 2**; **Supplementary Table 5**). Luminosity exhibited a strong positive correlation with hue (r=0.75) and a moderate positive correlation with chroma (r=0.36), suggesting coordinated regulation of fruit color development during ripening (**Figure 2**). Hue showed a significant positive correlation with chroma (r=0.51). Color-related attributes, including luminosity, hue, and chroma, exhibited a significant positive association, reflecting their coordinated contribution to fruit appearance during ripening (**Figure 2**).

**Figure 2:**
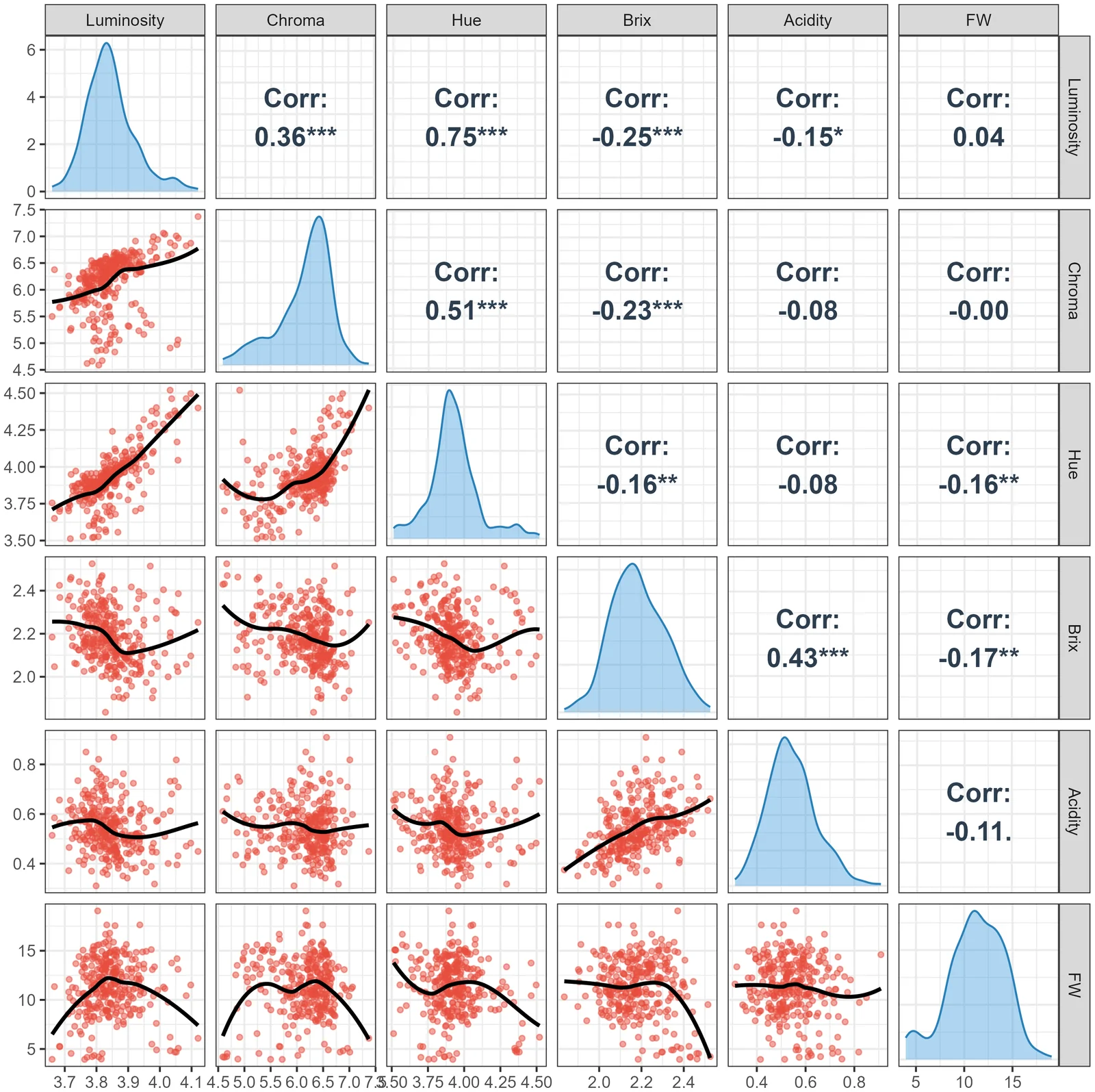
Pearson correlation coefficients among luminosity, hue, chroma, °Brix, acidity, and FW. Color intensity reflects the strength and direction of Pearson’s correlation coefficients calculated, with significance indicated by asterisks. *, **, *** Significance at 0.05, 0.01, 0.001 levels, respectively; ns: non-significant.

Brix showed a significant positive correlation with TA (r=0.43), indicating that fruits with greater sugar accumulation also tended to possess higher acid content (**Figure 2**). Conversely, Brix exhibited weak but significant negative correlations with luminosity (r=-0.25), hue (r=-0.16), and chroma (r=-0.23), suggesting potential trade-offs between fruit coloration and sugar accumulation (**Figure 2**). TA displayed a weak negative association with luminosity (r=-0.15). Fruit weight exhibited significant negative correlations with hue (r=-0.16) and brix (-0.17). Overall, the observed correlation patterns demonstrate that fruit quality traits are interconnected and may be regulated by overlapping physiological and genetic mechanisms.

### 3.2 Genome-wide association analysis

Following quality control, a total of 4,618 high-quality SNPs distributed across the 12 tomato chromosomes were retained for GWAS. Principal component analysis (PCA) showed that PC1 and PC2 explained 5.88% and 1.74% of the total genetic variation, respectively (**Supplementary Figure 1**). Population structure, phylogenetic relationships, and genome-wide LD were characterized for the diversity panel. Population structure analysis revealed genetic differentiation among the accessions, with the inferred groups (K = 3) showing varying levels of admixture (**Supplementary Figure 1**). The phylogenetic analysis (Q1-Q3) was consistent with the population-structure results and illustrated the genetic relationships among accessions within the diversity panel (**Supplementary Figure 1**). Genome-wide linkage disequilibrium declined with increasing physical distance between markers, providing an estimate of the extent of LD across the tomato genome (**Supplementary Figure 1**). Together, these analyses provide an overview of the genetic structure and relatedness of the diversity panel and were considered in the interpretation of the GWAS results.

To ensure robust identification of loci and minimize model-specific bias, seven GWAS models were implemented in GAPIT v4.1, including GLM, MLM, CMLM, SUPER, MLMM, FarmCPU, and BLINK. Overall, 15 significant non-redundant MTAs were detected for luminosity, hue, chroma, soluble solids content (°Brix), and FW, whereas no significant MTAs were identified for titratable acidity under the significance threshold of P ≤ 1×10^-5^ (**Table 3**) (**Figures 3**–**5**; **Supplementary Figure 2**). Quantile-quantile (Q-Q) plots indicated adequate control of population structure and relatedness, and Manhattan plots revealed distinct association peaks distributed across multiple chromosomes (**Figures 3**, **4**; **Supplementary Figure 2**).

**Table 3:** Significant MTAs identified by GWAS for fruit quality traits using multiple GWAS models in GAPIT 4.1.

S.No	SNP marker ID	Chr	Position (Mb)	Trait (Env)	Model	P-value	MAF	Effect size	R² (%)
1	SL4.0ch01_10984310	1	10984310	FW (Kansas); FW (NYC)	BLINK	9.86E-06	0.07	-1.66	6.3
2	SL4.0ch01_10993956	1	10993956	Hue (Kansas); Hue (NYC)	FARMCPU	7.91E-06	0.05	0.1	7.10 - 14.15
3	SL4.0ch01_34862987	1	34862987	FW (Kansas); FW (NYC)	FARMCPU	6.13E-06	0.15	0.97	3.43 - 5.77
4	SL4.0ch01_34927012	1	34927012	FW (Kansas); FW (NYC)	BLINK	4.42E-06	0.09	1.38	13.14
5	SL4.0ch01_55672077	1	55672077	Brix (Kansas); Brix (NYC)	FARMCPU	2.66E-06	0.23	-0.03	1.94 - 2.44
6	SL4.0ch03_12722521	3	12722521	Chroma (NYC); Hue (Kansas); Hue (NYC); Luminosity (Kansas); Luminosity (NYC)	BLINK, CMLM, FARMCPU, GLM, MLM, MLMM, SUPER	3.87E-09	0.05	-0.36 to -0.06	0.00 - 53.86
7	SL4.0ch04_8549	4	8549	Brix (Kansas); Brix (NYC)	FARMCPU	5.98E-06	0.09	-0.05	0.00 - 4.50
8	SL4.0ch05_5078214	5	5078214	Brix (NYC); FW (NYC)	CMLM, FARMCPU, GLM, MLM, SUPER	3.32E-09	0.06	-0.08 to +2.33	0.00 - 12.71
9	SL4.0ch05_5080334	5	5080334	Brix (NYC)	CMLM, GLM, MLM, SUPER	1.58E-07	0.07	-0.07 to -0.07	–
10	SL4.0ch05_5080391	5	5080391	Brix (Kansas); Brix (NYC); FW (NYC)	CMLM, FARMCPU, GLM, MLM, MLMM, SUPER	9.36E-13	0.07	-0.09 to +1.84	0.00 - 38.99
11	SL4.0ch05_5080764	5	5080764	Brix (NYC); FW (Kansas); FW (NYC)	BLINK, CMLM, FARMCPU, GLM, MLM, MLMM, SUPER	6.64E-17	0.05	-0.09 to +3.36	0.00 - 52.88
12	SL4.0ch05_5082109	5	5082109	Brix (NYC)	CMLM, GLM, MLM, SUPER	3.70E-08	0.06	-0.08 to -0.08	–
13	SL4.0ch09_68462831	9	68462831	FW (Kansas); FW (NYC)	FARMCPU	2.58E-06	0.04	1.42	13.07 - 33.66
14	SL4.0ch10_56518985	10	56518985	Chroma (NYC)	MLMM	1.47E-06	0.04	0.42	–
15	SL4.0ch12_36767446	12	36767446	Luminosity (NYC)	GLM, MLM, SUPER	8.44E-06	0.19	0.03	–

Kansas - Multi-step multi-locus framework and NYC - advanced iterative refinement layer.

**Figure 3:**
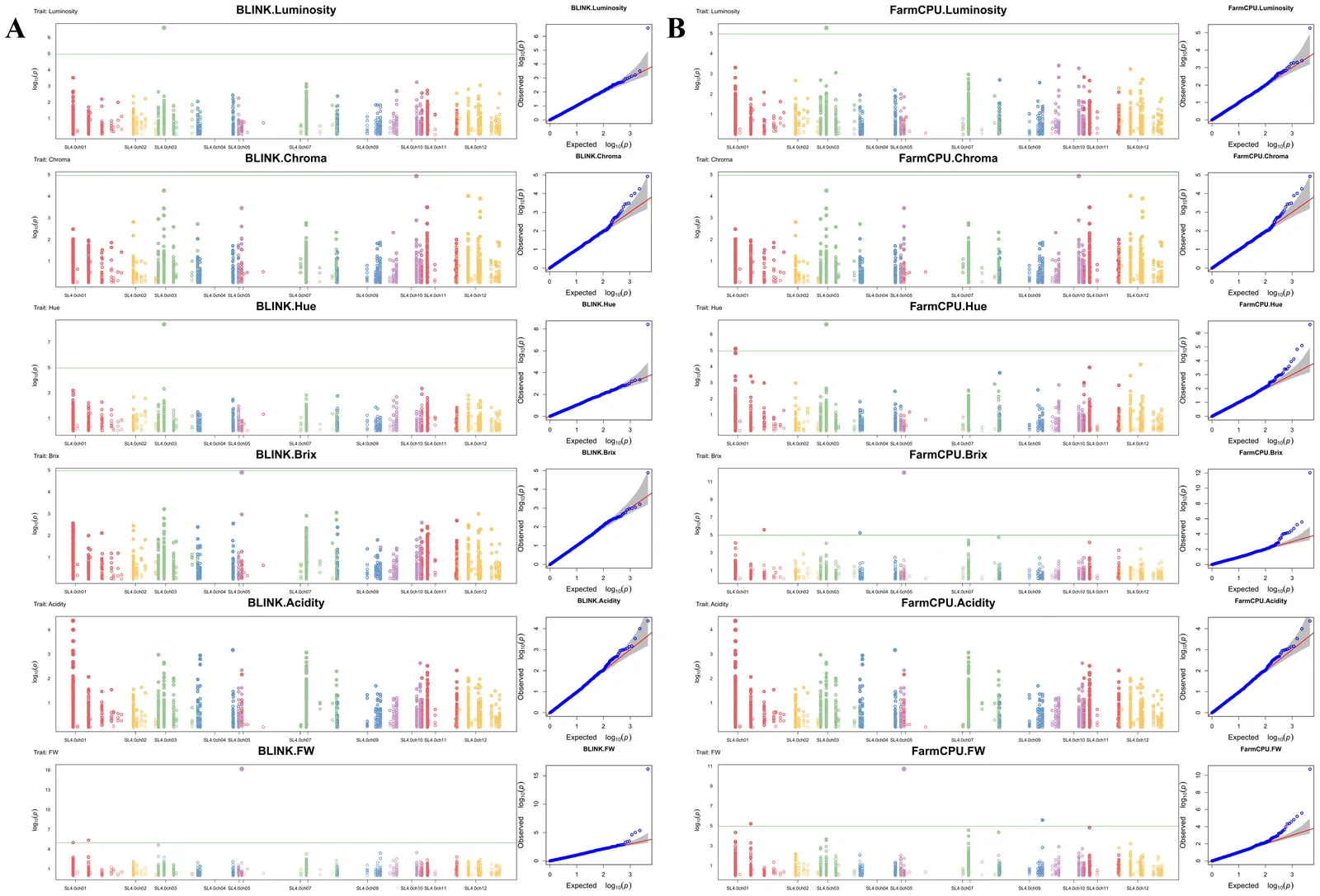
Genome-wide significant MTAs identified for tomato fruit quality traits using BLINK **(A)** and FarmCPU **(B)** models. Manhattan and quantile-quantile (Q-Q) plots for fruit quality traits. Each point represents one SNP tested across the genome. The horizontal threshold line indicates the significance cutoff of marker-trait associations (P ≤ 1×10^-5^). The plots compare observed and expected test statistics and show the extent to which population structure and relatedness were controlled across models.

**Figure 4:**
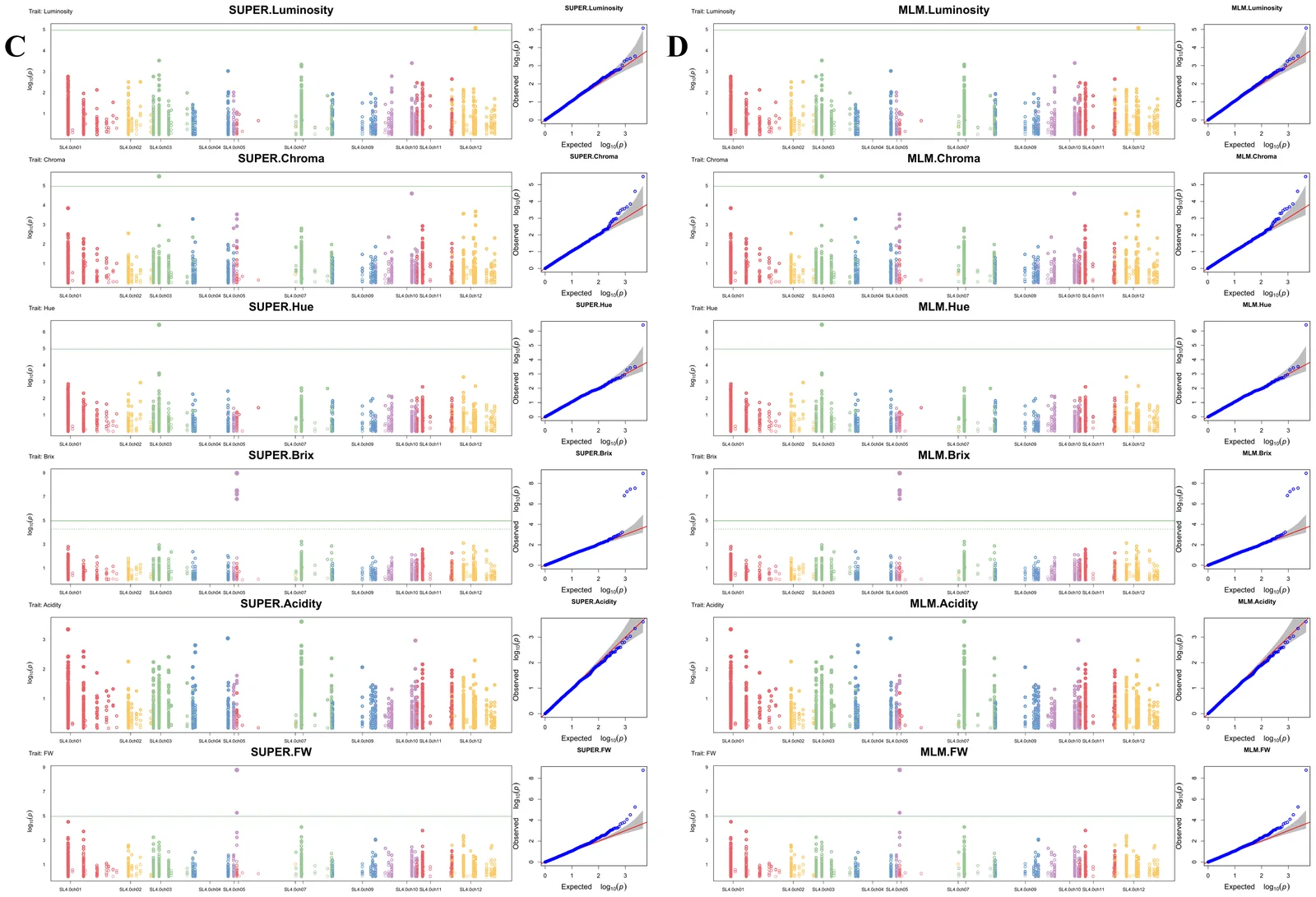
Genome-wide significant MTAs identified for tomato fruit quality traits using SUPER **(C)** and MLM **(D)** models. Manhattan and quantile-quantile (Q-Q) plots for fruit quality traits. Each point represents one SNP tested across the genome. The horizontal threshold line indicates the significance cutoff of marker-trait associations (P ≤ 1×10^-5^). The plots compare observed and expected test statistics and show the extent to which population structure and relatedness were controlled across models.

**Figure 5:**
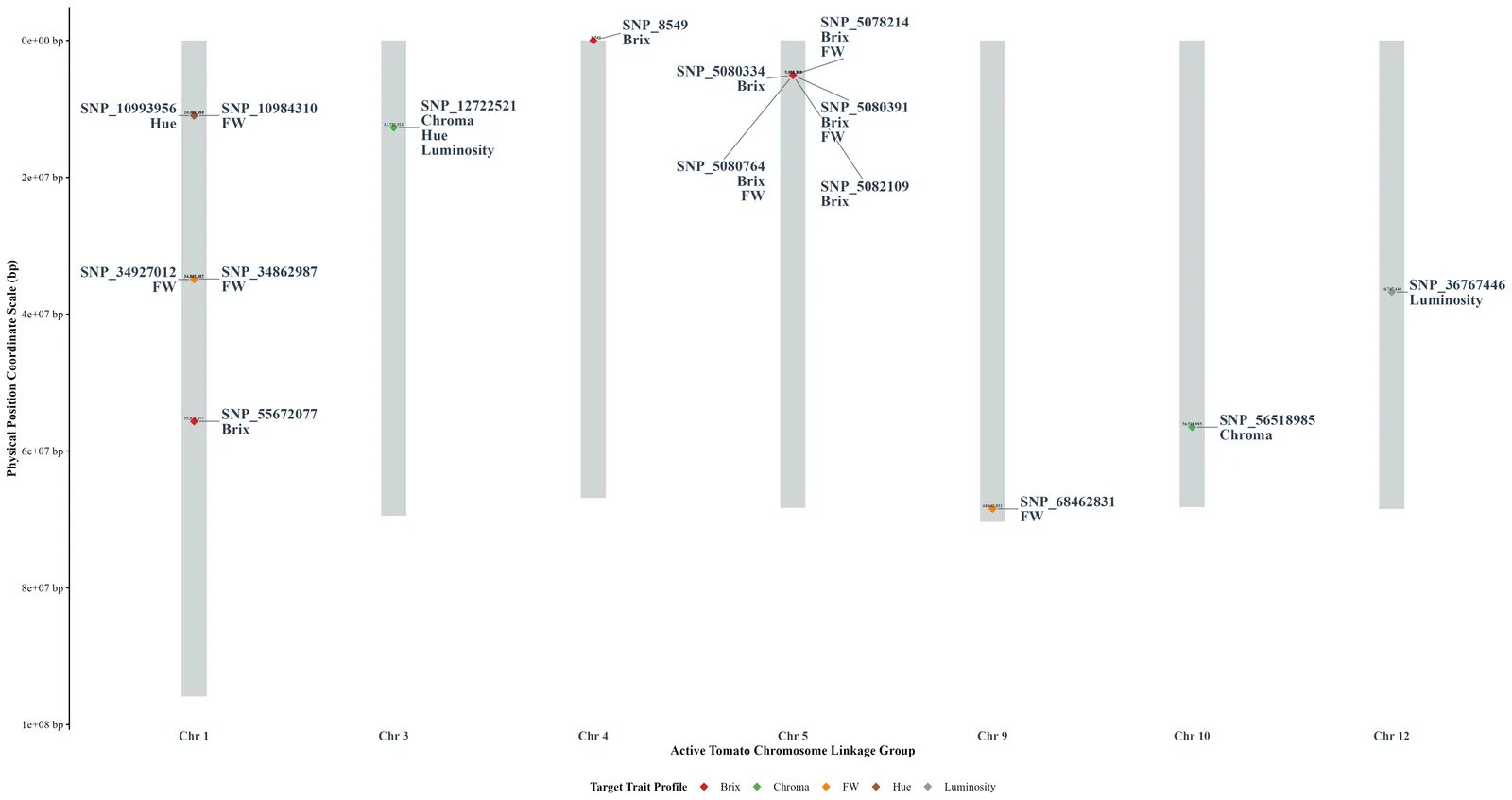
Distribution of significant marker-trait associations for fruit quality traits across the 12 tomato chromosomes.

For fruit color-related traits, significant MTAs were detected on chromosomes 1, 3, 10, and 12 (**Table 3**). The most prominent locus was SNP SL4.0ch03_12722521 on chromosome 3 associated with hue, luminosity, and chroma across multiple models, including BLINK, CMLM, FarmCPU, GLM, MLM, MLMM, and SUPER (**Table 3**) (**Figures 3**–**5**; **Supplementary Figure 2**). This locus showed an exceptionally strong association (P = 3.87 × 10^-9^) and explained up to 53.86% of phenotypic variation (PVE). The co-localization of multiple color-related traits at the same genomic region suggests either pleiotropic effects or tightly linked genes involved in pigment accumulation and fruit ripening. Hue was additionally associated with SNP SL4.0ch01_10993956 on chromosome 1 by the FarmCPU model (P = 7.91 × 10^-6^). This locus explained between 7.10 and 14.15% of PVE, indicating a moderate yet stable genetic contribution to fruit color development. For chroma, an additional significant locus (SL4.0ch10_56518985) was detected on chromosome 10 by the MLMM model (**Table 3**). Luminosity exhibited another significant association on chromosome 12 (SL4.0ch12_36767446), identified by GLM, MLM, and SUPER models (**Table 3**).

For °Brix, multiple MTAs were detected on chromosomes 1, 4, and 5, with chromosome 5 harboring most stable associations, highlighting this chromosome as a major genomic region controlling fruit sugar accumulation (**Table 3**; **Figures 3**–**5**; **Supplementary Figure 2**). Five significant SNPs, SL4.0ch05_5078214, SL4.0ch05_5080334, SL4.0ch05_5080391, SL4.0ch05_5080764, and SL4.0ch05_5082109, were repeatedly identified by multiple GWAS models, including CMLM, GLM, MLM, SUPER, FarmCPU, MLMM, and BLINK (**Table 3**). Among these, SNP SL4.0ch05_5080391 and SNP SL4.0ch05_5080764 exhibited exceptionally strong associations with °Brix and FW, with P-values reaching 9.36 × 10^−13^ and 6.64 × 10^−17^, respectively. These loci explained up to 38.99% and 52.88% of PVE, respectively. This chromosome 5 region also overlapped with fruit weight, suggesting a possible shared genomic region influencing both fruit composition and fruit size (**Table 3**; **Figure 5**).

Fruit fresh weight exhibited the most significant associations, reflecting its complex quantitative inheritance (**Table 3**). Significant MTAs were identified on chromosomes 1, 5, and 9 (**Table 3**; **Figures 3**–**5**; **Supplementary Figure 2**). SNP SL4.0ch05_5080764, located on chromosome 5, represented the most significant association for FW, detected by multiple GWAS models (**Table 3**). This locus explained up to 52.88% of PVE, suggesting the presence of a major genomic region controlling FW. An additional stable association was detected on chromosome 9 (SL4.0ch09_68462831), explaining 13.07-33.66% of PVE. Two additional loci, SL4.0ch01_34862987 and SL4.0ch01_34927012, were detected by FarmCPU and BLINK, respectively, explaining up to 5.77% and 13.14% of PVE, respectively. This chromosome 5 region also overlapped with °Brix in some cases, suggesting a possible shared genomic region influencing both fruit composition and fruit size. These stable MTAs provide valuable genomic resources for candidate gene identification, functional validation, and future marker-assisted breeding aimed at improving tomato fruit quality.

### 3.3 Candidate gene identification associated with fruit quality traits

To identify putative genes underlying the detected marker-trait associations (MTAs), genomic regions spanning ±50 kb around each significant SNP were examined using the tomato reference genome annotation. Genes located within the defined genomic intervals surrounding significant SNPs were considered putative candidate genes based on their genome annotations and predicted functions. These annotations provide hypotheses for future functional studies. Across all significant MTAs, a total of 79 annotated genes were detected (**Supplementary Table 6**). These genes encode proteins involved in diverse biological processes, including carbohydrate metabolism, transcriptional regulation, membrane-associated signaling, protein degradation, intracellular transport, defense responses, and several proteins of unknown function, suggesting that multiple biological pathways contribute to the genetic regulation of tomato fruit quality (**Supplementary Table 6**).

Among the identified MTAs, the genomic region surrounding SNP SL4.0ch09_68462831, on chromosome 9, associated with FW, contained multiple annotated genes, including an unknown protein, a survival motor neuron containing protein, sec14p-like phosphatidylinositol transfer family protein, sucrose synthase, and a plant UBX domain-containing protein 11 (**Supplementary Table 6**). Although some of these annotations are broad, the presence of sucrose synthase, sec14p-like phosphatidylinositol transfer family protein, and UBX domain-containing protein is noteworthy, as these proteins are often involved in regulating sink strength, ubiquitin-mediated protein turnover, developmental regulation, and stress-related signaling, all of which can affect fruit growth and size.

The highly significant MTA on chromosome 3 (SL4.0ch03_12722521), which was consistently associated with luminosity, hue, and chroma across multiple GWAS models, contained several nearby genes currently annotated as unknown proteins (**Supplementary Table 6**). Despite the absence of functional annotation, the stability of this locus across statistical models indicates that it represents a promising genomic region controlling fruit pigmentation and color-related traits. On chromosome 1, SNPs associated with hue were primarily located near genes encoding plastid transcriptionally active protein 3 (pTAC3) and several uncharacterized proteins (**Supplementary Table 6**). For chroma, the SNP SL4.0ch10_56518985 region on chromosome 10 contained several chitinase-related annotations, including chitinase and chitinase Z15140 (**Supplementary Table 6**).

Significant loci (SL4.0ch05_5078214, SL4.0ch04_8549, SL4.0ch05_5082109), associated with °Brix on chromosomes 4 and 5, co-localized with the genes encoding Phosphatidylinositol 4-kinase gamma 7, bHLH transcription factor 040, phosphoglucosamine mutase family proteins, metalloendoproteinase 1, aminotransferase-like, plant mobile domain family protein, and several proteins (**Supplementary Table 6**). These genes represent potential candidates involved in carbohydrate metabolism and fruit quality development.

Overall, candidate gene analysis identified numerous biologically relevant genes within significant GWAS intervals, although many neighboring genes remain uncharacterized. The abundance of genes with unknown annotations highlights the complexity of the genetic architecture controlling fruit quality traits and suggests that additional transcriptomic and functional genomic analyses will be necessary to identify the causal genes underlying these associations.

### 3.4 Allelic effects of significant MTAs

The allelic effect patterns support the biological relevance of the identified MTAs. Allelic effects were estimated using the transformed trait values used in the statistical and GWAS analyses; therefore, the means presented for individual allelic classes are on the transformed scale and should not be directly compared with the ranges reported on the original scale. For FW, multiple SNPs showed substantial allelic effects across the association panel. At SNP SL4.0ch05_5080764 on chromosome 5, one of the largest allelic effects was detected in this study (**Figure 6**; **Supplementary Table 7**). Accessions possessing the C allele produced fruits averaging 11.51 ± 2.64 g, whereas the alternative T allele was associated with substantially smaller fruits (4.47 ± 0.43 g), representing a difference of 7.04 g. A similar trend was observed for SNP SL4.0ch05_5078214, where the major allele was associated with significantly greater fruit weight than the alternative allele (**Figure 6**; **Supplementary Table 7**). SNP SL4.0ch01_34927012 on chromosome 1 for FW, where accessions carrying the T allele produced significantly heavier fruits (11.41 ± 2.86 g) than those carrying the A allele (7.84 ± 3.14 g).

**Figure 6:**
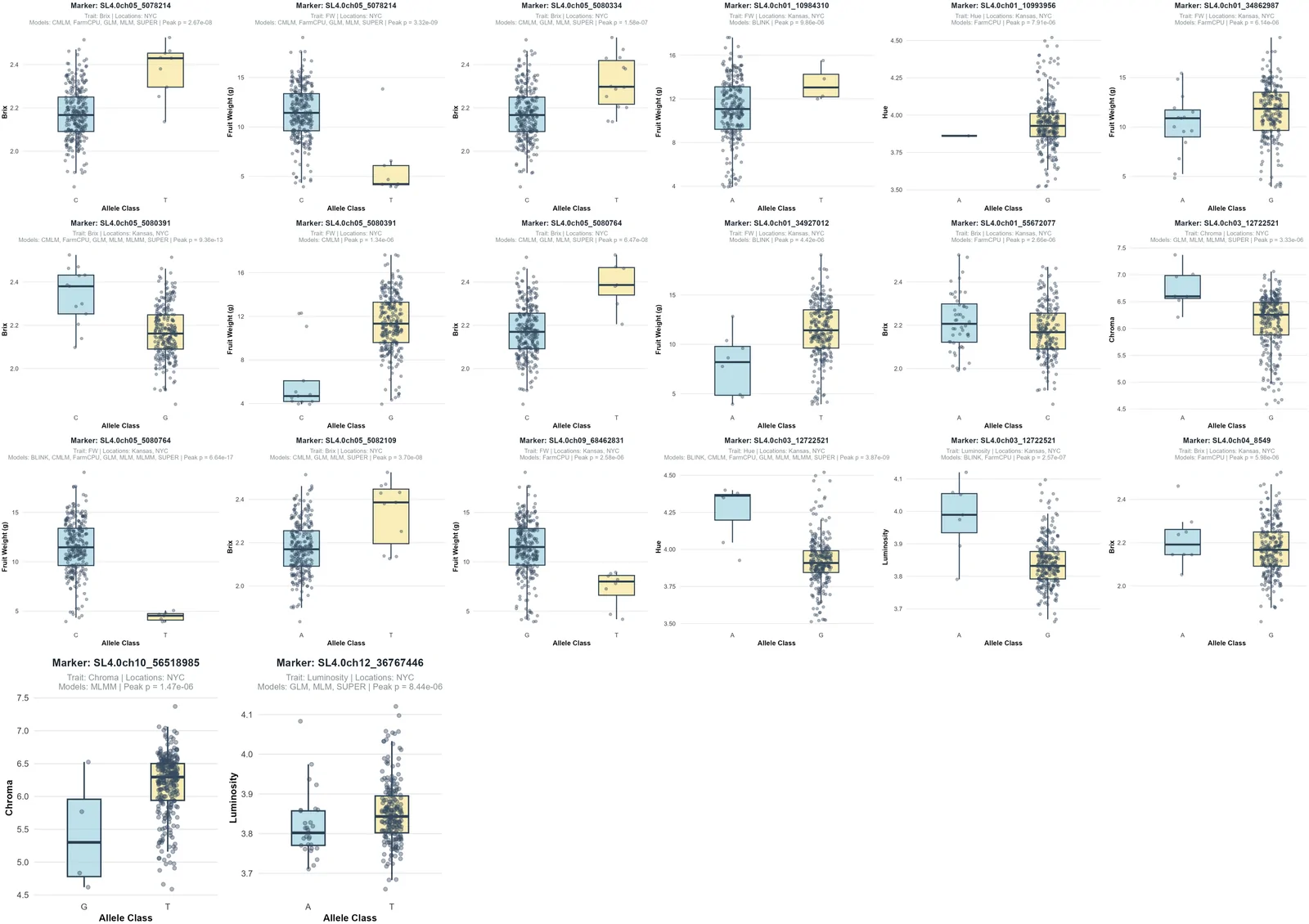
Allelic distribution and phenotypic effects of SNPs associated with fruit quality traits. For each MTA, the mean value is shown by allele class, highlighting favorable and unfavorable alleles for luminosity, hue, chroma, °Brix, and FW. Values shown represent means ± SD on the transformed scale used for statistical analysis.

Several loci exhibited pleiotropic effects on both FW and Brix (**Figure 6**; **Supplementary Table 7**). Notably, SNP SL4.0ch05_5080391 on chromosome 5 was associated with both traits. Accessions carrying the C allele displayed significantly higher soluble solids (2.34 ± 0.13°Brix) than those carrying the G allele (2.17 ± 0.12°Brix), while the same allele was associated with markedly reduced fruit weight (6.26 ± 3.26 g versus 11.41 ± 2.60 g) (**Figure 6**). Similarly, SNP SL4.0ch05_5080764 demonstrated contrasting allelic effects, where the allele conferring increased fruit weight was associated with lower soluble solids, reflecting the commonly observed inverse relationship between fruit size and sugar concentration (**Figure 6**; **Supplementary Table 7**).

For °Brix, several loci produced consistent allelic differences. At SNP, SL4.0ch01_55672077, the A allele resulted in significantly higher °Brix values than the C allele, whereas SNPs, SL4.0ch05_5078214, SL4.0ch05_5080334, SL4.0ch05_5080391, and SL4.0ch05_5082109 each exhibited significant increases in soluble solids associated with their favorable alleles (**Figure 6**; **Supplementary Table 7**). Although the magnitude of allelic effects for °Brix was relatively modest (0.15-0.22°Brix), these differences were highly significant and reproducible across environments, highlighting their potential utility for marker-assisted improvement of fruit sweetness.

Among color-related traits, SNP SL4.0ch03_12722521 on chromosome 3 displayed the strongest and most consistent allelic effects (**Figure 6**; **Supplementary Table 7**). Accessions carrying the minor A allele exhibited significantly greater hue (4.26 ± 0.19), luminosity (3.98 ± 0.11), and chroma (6.75 ± 0.38) compared with accessions possessing the G allele (**Figure 6**; **Supplementary Table 7**). These consistent allelic responses across three independent color parameters suggest that this locus may represent a major genomic region regulating fruit pigmentation and color development. In contrast, SNP SL4.0ch10_56518985 was specifically associated with chroma, where the T allele produced significantly higher chroma values than the alternative allele, confirming its role in determining fruit color intensity (**Figure 6**; **Supplementary Table 7**).

The luminosity-associated SNP, SL4.0ch12_36767446, showed a comparatively smaller but statistically significant allelic effect. Accessions carrying the T allele exhibited slightly higher luminosity values than those carrying the A allele, indicating that even loci with relatively small phenotypic effects may contribute to the polygenic regulation of fruit appearance (**Figure 6**; **Supplementary Table 7**).

Collectively, the allelic effect analyses confirmed that several significant MTAs identified through GWAS were associated with clear phenotypic differences among allelic classes. The consistency of these effects across different GWAS models further supports the robustness of the detected MTAs. These favorable alleles provide valuable molecular resources for marker-assisted selection and pyramiding of superior fruit quality alleles in tomato breeding programs.

## 4 Discussion

Fruit quality is one of the most complex breeding targets in tomato because it is determined by the combined effects of appearance, flavor, nutritional composition, and fruit size, all of which are controlled by numerous loci and are strongly influenced by environmental conditions (Beckles, 2012; Klee and Tieman, 2013; Lupi et al., 2019). In the present study, the integration of multi-season phenotyping, BLUE-based trait estimation, skim sequencing-derived SNP markers, and multiple GWAS models enabled a comprehensive dissection of the genetic architecture underlying six major fruit quality traits, including luminosity, hue, chroma, soluble solids content (°Brix), titratable acidity (TA), and FW. The success of GWAS depends largely on the extent of phenotypic variation present within the association panel (Korte and Farlow, 2013). In this study, a wide range of phenotypic variation was observed among 305 tomato accessions for luminosity, hue, chroma, °Brix, TA, and FW, confirming the existence of substantial genetic diversity in the association panel for fruit quality improvement (**Table 1**). Similar patterns of phenotypic variation have been widely reported in tomato diversity panels and reflect the complex genetic architecture underlying fruit quality traits (Kim et al., 2021; Liu et al., 2023b; Kayikci et al., 2025).

Broad-sense heritability (H^2^) provides valuable information regarding the relative contribution of genetic and environmental effects to phenotypic variation (Robinson et al., 1949). High heritability of fruit weight, hue, luminosity, chroma, and °Brix indicates that a substantial proportion of the observed phenotypic variation was attributable to genetic differences among accessions (Xu et al., 2013; Kim et al., 2021) (**Supplementary Table 4**). In contrast, the moderate heritability of acidity reflects the more complex biology of flavor-related traits (**Supplementary Table 4**). These traits are influenced by sugar metabolism, organic acid accumulation, ripening stage, and environmental fluctuations, which often reduce the proportion of phenotypic variance explained by genotype alone (Gautier et al., 2008; Xu et al., 2013; Kim et al., 2021). Similar trends have been reported in previous tomato studies, where fruit color traits generally exhibit higher heritability than biochemical characteristics associated with flavor (Xu et al., 2013; Kim et al., 2021). From a breeding perspective, these findings suggest that selection for fruit appearance and size can be achieved with relatively high efficiency, whereas improvement of sweetness and acidity requires multi-environment and multiple years evaluation to ensure stable genetic gain.

The trait correlations observed in this study provide additional insights into the biological relationships among fruit quality traits. The strong positive correlation between luminosity and hue (r=0.75) and the positive association of hue with chroma reflect the coordinated regulation of fruit pigmentation during ripening (**Figure 2**). This is consistent with the idea that fruit appearance is not governed by isolated traits but by coordinated changes in pigment accumulation, chloroplast-to-chromoplast transition, and ripening progression (Phan et al., 2019; Kayikci et al., 2025). In practical terms, selection for one color attribute may indirectly influence the others, which is useful for breeding programs targeting visual quality and market preference. The identification of common genomic regions controlling these traits further supports the existence of shared genetic pathways regulating fruit coloration. The positive correlation between °Brix and acidity (r=0.43) suggests that sugar and organic acid metabolism are partially coordinated during fruit maturation (**Figure 2**). Although sweetness and acidity jointly determine flavor intensity, their genetic regulation is highly complex because both traits are influenced by carbon partitioning, respiratory metabolism, and environmental conditions (Gautier et al., 2008; Ruggieri et al., 2014). The negative correlations of °Brix with luminosity, hue, and chroma may indicate physiological trade-offs between pigment accumulation and carbohydrate metabolism during fruit development (**Figure 2**). Likewise, the negative association between fruit weight and both hue and °Brix agrees with the commonly observed inverse relationship between fruit size and soluble solids concentration (Phan et al., 2019; Kayikci et al., 2025) (**Figure 2**). Larger fruits frequently exhibit lower sugar concentration because of dilution effects associated with increased water accumulation, whereas smaller-fruited genotypes generally possess higher soluble solids content (Ruggieri et al., 2014; Burato et al., 2025). These correlations highlight potential challenges in simultaneously improving fruit size and flavor and emphasize the need for molecular breeding strategies capable of pyramiding favorable alleles for multiple traits.

Genome-wide association mapping using 4,618 high-quality SNPs identified 15 non-redundant MTAs with fruit quality traits across the tomato genome (**Table 3**). The use of multiple GWAS algorithms implemented in GAPIT v4.1 substantially increased confidence in detected associations by reducing model-specific bias and allowing validation of loci across independent statistical approaches (Korte and Farlow, 2013). Among the evaluated models, multi-locus methods including BLINK, FarmCPU, and MLMM generally detected a greater number of significant associations than conventional single-locus models while maintaining adequate control of false-positive signals (Liu et al., 2016; Huang et al., 2019). This observation is consistent with previous reports demonstrating that multi-locus GWAS approaches possess greater statistical power for detecting loci controlling complex quantitative traits because they simultaneously account for multiple associated markers while reducing confounding caused by population structure and kinship (Zhang et al., 2010; Runcie and Crawford, 2019).

One of the most notable findings of this study was the identification of SNP SL4.0ch03_12722521 on chromosome 3, which was consistently associated with luminosity, hue, and chroma across all GWAS models, including BLINK, CMLM, FarmCPU, GLM, MLM, MLMM, and SUPER (**Table 3**; **Figure 5**). The consistency of this locus across traits and statistical models strongly suggests that this genomic region represents a major determinant of fruit color variation in tomato. The relatively large proportion of phenotypic variance (up to 53.86%) explained by this marker further supports its potential value for marker-assisted breeding. In association panel, effect-size and PVE estimates can be sensitive to allele frequency, sampling variance, model specification, and the distribution of the phenotype. Therefore, the reported PVE values are interpreted as statistical estimates of marker-associated variation rather than direct estimates of the proportion of biological variance controlled by individual loci. Independent validation will be required to determine the stability and biological contribution of these loci. The chromosome 3 interval contained mostly unknown proteins, indicating that this may represent a novel region for tomato color biology (**Supplementary Table 6**). In high-quality association studies, such regions are often among the most interesting because they suggest previously unrecognized regulators of ripening-associated color change (Ballester et al., 2010; Kayikci et al., 2025). The allelic effects at this locus were directionally consistent with the phenotypic correlations, further supporting its biological relevance: the favorable allele shifted the color traits in a way that reflected stronger and more uniform ripening pigmentation (Kayikci et al., 2025) (**Figure 6**; **Supplementary Table 6**).

Fruit weight was associated with several genomic regions located on chromosomes 1, 5, and 9 (**Table 3**; **Figure 5**). Particularly noteworthy were SNP SL4.0ch05_5080391 and SNP SL4.0ch05_5080764 on chromosome 5, which exhibited highly significant associations across multiple GWAS models (**Table 3**). Their detection by BLINK, FarmCPU, MLMM, CMLM, GLM, MLM, and SUPER indicates exceptional stability and strongly supports their biological relevance (**Figures 3**, **4**; **Supplementary Figure 2**). Similarly, SNP SL4.0ch09_68462831 on chromosome 9 showed associations with FW, explaining between 13.07 and 33.66% of phenotypic variation, suggesting that multiple genomic regions collectively contribute to fruit weight. The region on chromosome 9 is especially noteworthy because it contains several candidate genes, including a sucrose synthase, Sec14p-like phosphatidylinositol transfer protein, a ppGpp synthetase-related gene, and a UBX domain-containing protein, as well as several uncharacterized genes (**Supplementary Table 6**). Among these, sucrose synthase is the most biologically intuitive candidate because of its role in sucrose cleavage and carbon partitioning during sink development, and is considered a key regulator of carbohydrate utilization in developing fruits (Barbosa et al., 2023). Enhanced sucrose metabolism increases the supply of UDP-glucose and fructose required for cell division, cell expansion, and starch biosynthesis, ultimately influencing fruit growth and biomass accumulation (Liu et al., 2023a). Previous studies have consistently reported that altered expression of sucrose synthase genes affects fruit size, yield components, and sink strength in tomato and several other crop species (Barbosa et al., 2023). Another noteworthy candidate identified in this region encodes a Sec14p-like phosphatidylinositol transfer family protein (**Supplementary Table 6**). Members of this protein family regulate phospholipid metabolism, vesicle trafficking, and membrane signaling pathways that are essential for cell expansion, intracellular transport, and organ development (Montag et al., 2023). The other annotated genes may influence cellular energy status, protein turnover, or developmental regulation, any of which could indirectly affect fruit expansion (Mousley et al., 2012; Hauvermale et al., 2022; Montag et al., 2023; Boniecka et al., 2025) (**Supplementary Table 6**). In practical terms, the favorable alleles at these fruit-weight loci were associated with measurable increases in fruit mass, confirming that the detected associations are not only statistically significant but also important for selection in both fresh-market and processing types.

Although chromosome 1 signals were surrounded mainly by unannotated proteins for FW, the presence of Plastid transcriptionally active 3 (PTA3) near SNP SL4.0ch01_10984310 is noteworthy (**Supplementary Table 6**). Plastid-related genes are increasingly recognized as important contributors to fruit development because plastids influence photosynthetic assimilation, carbon allocation, and developmental signaling during fruit growth (Kahlau and Bock, 2008; Ruggieri et al., 2014; Kim et al., 2021). Even when the precise causal variant is not obvious, the co-localization of fruit-weight signals with plastid-associated genes suggests that this region may affect source-sink balance or developmental regulation during fruit enlargement.

Soluble solids content, measured as °Brix, was controlled by a cluster of associations on chromosomes 1, 4, and especially 5 (**Table 3**). Among these, chromosome 5 was the most important genomic region, with SNPs SL4.0ch05_5078214, SL4.0ch05_5080391, SL4.0ch05_5080764, and SL4.0ch05_5082109 being repeatedly detected across different GWAS models (**Table 3**). This pattern indicates that chromosome 5 represents an important hotspot regulating sugar accumulation in tomato fruits. The candidate genes in this interval provide a biologically coherent explanation for trait architecture. The presence of an aminotransferase-like plant mobile domain protein, bHLH transcription factor 040, phosphatidylinositol 4-kinase gamma 7, oligopeptidase B, 3-methyladenine DNA glycosylase, and tetratricopeptide repeat-like proteins suggests that this locus may influence metabolic regulation, transcriptional control, signaling, and stress-responsive processes linked to sugar accumulation (**Supplementary Table 6**). The bHLH transcription factor is particularly interesting because transcription factors of this class are often central regulators of developmental and metabolic pathways, including carbohydrate-related processes (Kayikci et al., 2025; Martínez-Estrada et al., 2025; Shi et al., 2025). Likewise, phosphatidylinositol 4-kinase gamma 7 may influence vesicle trafficking and signaling events that affect fruit metabolism (Zhao and Xue, 2020). Although their direct roles in determining soluble solids content remain to be experimentally validated, these genes collectively suggest that sugar accumulation is controlled by a complex regulatory network rather than by a single metabolic pathway.

The allelic effects for chromosome 5 were especially informative. At several loci, the favorable allele increased °Brix while also reducing fruit weight, which mirrors the well-known biological trade-off between fruit size and sugar concentration in tomato (**Figure 6**; **Supplementary Table 7**). This pattern is not only expected but also highly relevant for breeding. The results suggest that improving flavor through higher soluble solids may require selecting alleles that increase sugar accumulation without disproportionately reducing fruit size (Kim et al., 2021; Kayikci et al., 2025). The fact that these chromosome 5 MTAs were detected across GWAS models strengthens their value as breeding targets (**Table 3**). Among them, SNP SL4.0ch05_5080764 was particularly notable because it affected both °Brix and fruit weight, implying either close linkage or pleiotropic control over carbon allocation and fruit development. Therefore, future breeding efforts may benefit from pyramiding favorable alleles from independent loci controlling sugar accumulation and fruit weight.

The chroma-associated locus on chromosome 10 contained multiple chitinase genes, including Chitinase and chitinase Z15140 (**Supplementary Table 6**). Although chitinases are traditionally associated with plant defense responses, increasing evidence indicates that they also participate in developmental processes, cell wall remodeling, and fruit ripening (Wu et al., 2001). Cell wall modification during ripening alters fruit surface properties and tissue organization, which may indirectly influence light reflection and color intensity. The favorable allele at SNP SL4.0ch10_56518985 increased chroma, indicating that this locus may be useful in breeding programs seeking improved external fruit appearance and stronger color saturation (**Figure 6**; **Supplementary Table 7**).

In addition, the candidate genes identified in this study were assigned based on physical proximity to significant SNPs. Functional validation through transcriptome analysis, expression profiling, gene editing, or mutant characterization will be necessary to establish causal relationships between these genes and fruit quality traits. Future studies integrating GWAS with transcriptomics, metabolomics, and genomic prediction will provide a more comprehensive understanding of the molecular networks governing tomato fruit quality. Such integrative approaches are expected to accelerate the discovery of causal genes and facilitate their direct deployment in modern breeding programs.

## 5 Conclusion

Overall, this study demonstrated that genome-wide association mapping using a diverse tomato panel, two years of phenotypic evaluation, BLUE-based phenotypic estimates, and multiple GWAS models is an effective strategy for dissecting the genetic architecture of fruit quality traits. Several stable MTAs were consistently identified across statistical models, particularly for fruit color, soluble solids content, and fruit weight, providing valuable genomic resources for tomato improvement. Candidate genes involved in carbohydrate metabolism, membrane signaling, defense-related processes, and fruit development provide promising targets for fine mapping, functional characterization, and validation in independent populations. Collectively, these findings expand the current understanding of the genetic basis of tomato fruit quality and provide valuable genomic resources for marker-assisted selection, genomic prediction, and the development of high-quality tomato cultivars.

This project was supported by the USDA Agriculture and Food Research Initiative, National Institute of Food and Agriculture (NIFA) (2024-51181-43464, 2023-67013-39616, TEX0-1-9618), SARE 2023-38640-39572, and Vegetable and Fruit Improvement Center, Texas A&M seed grants.

## Data Availability

The original contributions presented in the study are included in the article/**Supplementary Material**, further inquiries can be directed to the corresponding author/s.
